# Adjustment of the GRACE Risk Score by Monocyte to High-Density Lipoprotein Ratio Improves Prediction of Adverse Cardiovascular Outcomes in Patients With Acute Coronary Syndrome Undergoing Percutaneous Coronary Intervention

**DOI:** 10.3389/fcvm.2021.755806

**Published:** 2022-01-26

**Authors:** Xiaoteng Ma, Kangning Han, Lixia Yang, Qiaoyu Shao, Qiuxuan Li, Zhijian Wang, Yueping Li, Fei Gao, Zhiqiang Yang, Dongmei Shi, Yujie Zhou

**Affiliations:** Department of Cardiology, Beijing Anzhen Hospital, Capital Medical University, Beijing, China

**Keywords:** monocyte to high-density lipoprotein cholesterol ratio, GRACE risk score, acute coronary syndrome, percutaneous coronary intervention, cardiovascular outcomes

## Abstract

**Background and Aims:**

The monocyte to high-density lipoprotein cholesterol ratio (MHR), a novel marker for inflammation and lipid metabolism, has been demonstrated to be associated with poor prognosis in many patient populations. However, the prognostic influence of MHR in patients with acute coronary syndrome (ACS) undergoing percutaneous coronary intervention (PCI) is poorly understood. Here, we sought to investigate the relationship between MHR and adverse cardiovascular (CV) outcomes in such patients and determine whether MHR could improve the GRACE risk score based prognostic models.

**Methods and Results:**

MHR was applied to 1,720 patients with ACS undergoing PCI who were admitted to our CV center from June 2016 to November 2017. These patients were stratified into three groups according to MHR tertiles. The relationship between MHR and the primary endpoint (overall death, non-fatal stroke, non-fatal myocardial infarction, or unplanned repeat revascularization) was examined by Cox proportional hazards regression analysis. During a median follow-up of 31 months, 353 patients had at least one primary endpoint event. Compared with those in the lowest MHR tertile, patients in the middle and highest tertiles [adjusted HR: 1.541 (95% CI: 1.152–2.060) and 1.800 (95%CI: 1.333–2.432), respectively], had a higher risk of the primary endpoint. The addition of MHR has an incremental effect on the predictive ability of the GRACE risk score for the primary endpoint (cNRI: 0.136, *P* < 0.001; IDI: 0.006, *P* < 0.001).

**Conclusion:**

MHR was independently and significantly associated with adverse CV outcomes in ACS patients who underwent PCI and improved the predictive ability of the GRACE risk score based prognostic models.

**Registration Number:**

http://www.chictr.org.cn/hvshowproject.aspx?id=21397; ChiCTR1800017417.

## Introduction

Coronary artery disease (CAD), as one of the leading causes of death in humans, is mainly caused by atherosclerosis (1). The formation of atherosclerotic plaque is known to be characterized by the accumulation of low-density lipoprotein cholesterol (LDL-C) and monocyte-derived macrophages in the arterial wall (2). Further plaque rupture can expose subendothelial collagen and cause an inflammatory response, which leads to platelet activation and coagulation cascade, resulting in thrombus formation (3). Thrombus can cause partial or complete occlusion of the coronary artery, leading to acute coronary syndrome (ACS) (4).

The number of circulating blood monocytes is closely associated with the formation and expansion of atherosclerosis in both human and animal models (5, 6). Monocytes can secrete enzymes that degrade extracellular matrix, contributing to the rupture of plaque underlying ACS (7). High-density lipoprotein (HDL), a cardioprotective factor, exhibits anti-atherosclerotic properties by neutralizing the pro-inflammatory and pro-oxidative effects of monocytes via inhibiting macrophage migration and LDL-C oxidation and mediating efflux of cholesterol from cells (8, 9). Of note, a recent Mendelian randomization study showed that low HDL cholesterol (HDL-C) was associated with high monocyte count (10). Therefore, the combination of monocyte and HDL-C may reflect the inflammatory and metabolic process of atherosclerosis better than the individual monocyte or HDL-C (9). The monocyte to HDL-C ratio (MHR) has been demonstrated to be associated with poor prognosis in many patient populations (11–19). The study of Cetin et al. including 2,661 patients with ACS showed that MHR was an independent predictor of CAD severity and future cardiovascular (CV) events (19). Nevertheless, the prognostic influence of MHR in patients with ACS undergoing percutaneous coronary intervention (PCI) has not been adequately studied and, importantly, few studies have evaluated the incremental value of adding MHR to the GRACE (Global Registry of Acute Coronary Events) risk score based prognostic models.

The objective of the present study was to investigate the relationship between MHR and CV outcomes in patients with ACS undergoing PCI, and then determine whether MHR could improve the GRACE risk score based prognostic models.

## Methods

### Study Population

This study was a retrospective analysis derived from a prospective observational study that sought to identify novel risk factors for adverse CV events in patients with ACS undergoing PCI who were admitted to our CV center from June 2016 to November 2017 (20). ACS was diagnosed according to current guidelines and was classified into unstable angina (UA), non-ST-segment elevation myocardial infarction (NSTEMI), and ST-segment elevation myocardial infarction (STEMI) (21). The exclusion criteria of this study included past coronary artery bypass grafting, history of rheumatism, infectious disease, niacin intake, and lack of follow-up data. Eventually, a total of 1,720 patients comprised the study population. This study was conducted under the Declaration of Helsinki and was approved by the local Ethics Committee. Since the present study was retrospective, the informed consent was waived.

### Data Collection

Data on demographics, medical history, and medication history were collected using a standard questionnaire. MHR on admission was calculated as monocyte count (× 10^6^/μl) divided by HDL-C levels (mg/dl). Body mass index (BMI) was calculated as body weight in kilograms divided by the square of body height in meters (kg/m^2^). Chronic kidney disease was defined as estimated glomerular filtration rate <60 ml/min/1.73 m^2^, calculated by the Chronic Kidney Disease Epidemiology Collaboration equation. Patients with blood pressure ≥140/90 mmHg or receiving anti-hypertensive treatments were considered as having hypertension. Dyslipidemia was diagnosed as total cholesterol > 5.17 mmol/l, triglycerides > 1.69 mmol/l, LDL-C > 3.36 mmol/l, HDL-C <1.03 mmol/l, and/or use of lipid-lowering drugs. Patients with the previous diagnosis of diabetes, fasting plasma glucose (FPG) ≥ 7.0 mmol/L, 2-h plasma glucose of oral glucose tolerance test ≥11.1 mmol/l or treated with hypoglycemic drugs were considered to have diabetes. Diagnosis of peripheral artery disease (PAD) was based on the ultrasound results and symptoms. Patients with previous ischemic stroke or transient ischemic attack were defined as cerebrovascular accident (CVA). The SYNTAX (Synergy between PCI with TAXUS and Cardiac Surgery) score and GRACE risk score were calculated for each patient.

### Follow Up and Outcomes

The follow-up time points were 1 month and every 6 months after discharge. The primary endpoint was defined as the composite of overall death, non-fatal stroke, non-fatal myocardial infarction (MI), and unplanned repeat revascularization. The hard endpoint was defined as the composite of cardiovascular death, non-fatal stroke, and non-fatal MI. Stroke was defined as an acute episode of focal or global neurological dysfunction caused by cerebral vascular injury because of infarction. MI was defined as cardiac enzymes above the upper limit of reference values accompanied by ischemic symptoms and/or electrocardiogram changes. If patients had multiple events during follow-up, the most severe endpoint event was selected for analysis (death > stroke > MI > revascularization). If more than one stroke, MI, or revascularization occurred, only the first event was analyzed.

### Statistical Analyses

The study population was stratified into three groups according to the tertiles of MHR on admission (T1: < 7.7; T2: 7.7–11.3; T3: > 11.3). Continuous variables were presented as mean ± standard deviation (SD) or median and interquartile range (IQR) for normal or non-normal distribution where *t*-test or Mann-Whitney *U*-test was used properly. Categorical variables were expressed as numbers and percentages where the Chi-square test (χ^2^ test) or Fisher's exact test was used accordingly. ANOVA or Kruskal–Wallis *H*-test was applied to analyze differences in continuous variables among three groups. Pearson correlation analysis was performed to evaluate the correlation between MHR and high-sensitivity C-reactive protein (hs-CRP), neutrophil to lymphocyte ratio (NLR), SYNTAX score, and GRACE risk score. Time-to-event curves stratified by MHR tertiles were drawn by the Kaplan–Meier method and were compared using log-rank tests. Cox proportional hazards regression analyses were used to determine the predictors of the primary endpoint. The MHR was analyzed in two ways: (1) as a categorical variable; and (2) as a continuous variable. Predictors of the incidence of the endpoint events identified through univariate analysis were also tested in a multivariate analysis. In the multivariate model, the following confounding factors were chosen because of their clinical importance and statistical significance in the univariate analysis: hs-CRP (continuous), sex, smoking, hypertension, diabetes, dyslipidemia, previous MI, previous PCI, previous CVA, PAD, type of ACS, GRACE risk score (continuous), SYNTAX score (continuous), complete revascularization, and use of aspirin, angiotensin converting enzyme inhibitors/angiotensin receptor blockers (ACEI/ARBs), and β-blockers at discharge. In order to avoid repeatedly adding the same or highly correlated variables to affect Cox model fitting, components of the GRACE risk score were not included in the multivariate Cox regression model which had included the GRACE risk score. Receiver operating characteristic (ROC) analysis was used to determine cut-off values of MHR to predict the occurrence of the primary endpoint and hard endpoint. Subgroup analyses stratified by sex, age, smoking, hypertension, diabetes, dyslipidemia, types of ACS, and medications at discharge were performed. The incremental predictive value of adding MHR to models with GRACE risk score was analyzed by calculating the increase in C-statistics, category-free continuous net reclassification improvement (cNRI) and integrated discrimination improvement (IDI).

The α level of significance was *P* < 0.05 two-sided. Statistical analyses were performed using SPSS software (version 26, SPSS Inc., Chicago, Illinois) and R software (version 4.1.0, R Foundation for Statistical Computing, Beijing, China).

## Results

Of the 1,720 patients (mean age 60 ± 10 years), 401 (23.3%) were female and 1,319 (76.7%) were male. The baseline characteristics according to the MHR tertiles are shown in Table 1. Patients with higher MHR tertiles were younger, and had lower levels of blood pressure, total cholesterol, HDL-C, and left ventricular ejection fraction (LVEF), but had higher levels of BMI, monocyte count, neutrophil count, lymphocyte count, NLR, hs-CRP, triglycerides, FPG, glycosylated hemoglobin, cardiac troponin I (cTnI), SYNTAX score, and GRACE risk score. Patients with higher MHR tertiles were more likely to be male, and had higher rates of smoking, diabetes, dyslipidemia, previous MI, PAD, heart failure, MI, left main and/or multivessel lesions, and chronic total occlusion, but had lower rates of complete revascularization. In correlation analysis, MHR was significantly and positively correlated with hs-CRP (*r* = 0.412, *P* < 0.001), neutrophil to lymphocyte ratio (*r* = 0.203, *P* < 0.001), SYNTAX score (*r* = 0.115, *P* < 0.001), and GRACE risk score (*r* = 0.226, *P* < 0.001).

**Table 1:** Baseline characteristics of the study population according to the MHR tertiles.

**Variables**	**T1: < 7.7**	**T2: 7.7–11.3**	**T3: > 11.3**	***P*** **-value**
MHR	5.9 (4.9–6.9)	9.3 (8.5–10.2)	14.1 (12.4–16.7)	<0.001
Age (years)	61 ± 9	60 ± 10	58 ± 11	<0.001
Male sex, *n* (%)	355 (62.7)	449 (77.4)	515 (89.7)	<0.001
BMI (kg/m^2^)	25.2 ± 2.9	25.7 ± 3.1	26.2 ± 3.1	<0.001
SBP (mmHg)	132 ± 16	131 ± 16	127 ± 17	<0.001
DBP (mmHg)	77 ± 11	77 ± 10	75 ± 11	0.001
**Risk factors**				
Smoking, *n* (%)	156 (27.6)	250 (43.1)	354 (61.7)	<0.001
Hypertension, *n* (%)	373 (65.9)	364 (62.8)	358 (62.4)	0.397
Diabetes, *n* (%)	246 (43.5)	303 (52.5)	241 (42.0)	0.001
Dyslipidemia, *n* (%)	379 (67.0)	468 (80.7)	528 (92.0)	<0.001
Previous MI, *n* (%)	88 (15.5)	110 (19.0)	132 (23.0)	0.006
Previous PCI, *n* (%)	109 (19.3)	118 (20.3)	114 (19.9)	0.899
Previous CVA, *n* (%)	26 (4.6)	34 (5.9)	40 (7.0)	0.230
CKD, *n* (%)	12 (2.1)	13 (2.2)	24 (4.2)	0.063
PAD, *n* (%)	43 (7.6)	66 (11.4)	67 (11.7)	0.041
Heart failure, *n* (%)	28 (4.9)	37 (6.4)	55 (9.6)	0.007
LVEF (%)	65 (61–68)	65 (60–68)	63 (58–67)	<0.001
**Clinical presentation**				
UA, *n* (%)	482 (85.2)	439 (75.7)	355 (61.8)	<0.001
NSTEMI, *n* (%)	56 (9.9)	78 (13.4)	86 (15.0)	0.031
STEMI, *n* (%)	28 (4.9)	63 (10.9)	133 (23.2)	<0.001
GRACE risk score	92 (73–110)	92 (77–123)	104 (77–144)	<0.001
**Laboratory results**				
Monocyte count (× 10^6^/μl)	260 (210–300)	360 (320–408)	500 (430–590)	<0.001
Neutrophil count (× 10^6^/μl)	3,365 (2,770–4,173)	3,970 (3,253–4,780)	4,670 (3,860–5,663)	<0.001
Lymphocyte count (× 10^6^/μl)	1,575 (1,328–1,903)	1,780 (1,440–2,190)	1,920 (1,540–2,380)	<0.001
NLR	2.1 (1.6–2.9)	2.3 (1.8–2.9)	2.4 (1.8–3.3)	<0.001
hs-CRP (mg/L)	0.88 (0.42–1.84)	1.46 (0.71–3.38)	2.29 (0.91–6.43)	<0.001
Total cholesterol (mg/dl)	166.0 ± 38.8	160.2 ± 36.9	154.8 ± 38.3	<0.001
LDL-C (mg/dl)	96.3 ± 33.2	94.1 ± 30.7	92.8 ± 30.0	0.165
HDL-C (mg/dl)	46.4 ± 8.7	39.2 ± 7.3	34.3 ± 6.7	<0.001
Triglycerides (mg/dl)	106.3 (78.8–151.5)	136.0 (95.7–192.9)	143.9 (105.2 ± 200.4)	<0.001
FPG (mg/dl)	111.6 ± 29.1	116.3 ± 32.0	115.1 ± 31.8	0.027
Glycosylated hemoglobin (%)	5.9 (5.5–6.9)	6.3 (5.6–7.3)	6.1 (5.6–7.2)	<0.001
cTnI (ng/ml)	0.00 (0.00–0.01)	0.00 (0.00–0.01)	0.01 (0.00–0.10)	<0.001
**Admission medical therapy**				
Aspirin, *n* (%)	395 (69.8)	435 (75.0)	432 (75.3)	0.062
P2Y12 inhibitors, *n* (%)	193 (34.1)	255 (44.0)	253 (44.1)	<0.001
Statins, *n* (%)	384 (67.8)	426 (73.4)	428 (74.6)	0.026
ACEI/ARBs, *n* (%)	161 (28.4)	175 (30.2)	155 (27.0)	0.491
β-blockers, *n* (%)	190 (33.6)	212 (36.6)	237 (41.3)	0.025
**Angiographic findings**				
Left-main and/or multivessel disease, *n* (%)	469 (82.9)	480 (82.8)	509 (88.7)	0.006
Chronic total occlusion, *n* (%)	105 (18.6)	116 (20.0)	142 (24.7)	0.027
Lesions with length >20 mm, *n* (%)	294 (51.9)	295 (50.9)	313 (54.5)	0.440
Bifurcation or trifurcation lesions, *n* (%)	434 (76.7)	434 (74.8)	430 (74.9)	0.715
SYNTAX score	19 (12–27)	20 (13–27)	22 (14–31)	<0.001
**Procedural results**				
Target vessel-LM, *n* (%)	19 (3.4)	18 (3.1)	20 (3.5)	0.935
Target vessel-LAD, *n* (%)	129 (22.8)	142 (24.5)	124 (21.6)	0.505
Target vessel-LCX, *n* (%)	71 (12.5)	85 (14.7)	67 (11.7)	0.300
Target vessel-RCA, *n* (%)	105 (18.6)	122 (21.0)	96 (16.7)	0.170
Complete revascularization, *n* (%)	377 (66.6)	378 (65.2)	302 (52.6)	<0.001
**Prescription at discharge**				
Aspirin, *n* (%)	564 (99.6)	574 (99.0)	566 (98.6)	0.178
Clopidogrel, *n* (%)	518 (91.5)	536 (92.4)	525 (91.5)	0.804
Ticagrelor, *n* (%)	48 (8.5)	44 (7.6)	49 (8.5)	0.804
Statins, *n* (%)	566 (100.0)	580 (100.0)	574 (100.0)	NA
ACEI/ARBs, *n* (%)	253 (44.7)	278 (47.9)	297 (51.7)	0.059
β-blockers, *n* (%)	373 (65.9)	397 (68.4)	441 (76.8)	<0.001

*MHR, monocyte to HDL-C ratio; BMI, body mass index; SBP, systolic blood pressure; DBP, diastolic blood pressure; CAD, coronary artery disease; MI, myocardial infarction; PCI, percutaneous coronary intervention; CVA, cerebrovascular accident; PAD, peripheral artery disease; LVEF, left ventricular ejection fraction; CKD, chronic kidney disease; UA, unstable angina; NSTEMI, non ST-segment elevation myocardial infarction; STEMI, ST-segment elevation myocardial infarction; GRACE, Global registry of acute coronary events; NLR, neutrophil to lymphocyte ratio; hs-CRP, high sensitive C-reactive protein; LDL-C, low-density lipoprotein-cholesterol; HDL-C, high-density lipoprotein-cholesterol; FPG, fasting plasma glucose; cTnI, cardiac troponin I; ACEI, angiotensin converting enzyme inhibitor; ARB, angiotensin receptor blocker; SYNTAX, Synergy between PCI with TAXUS and Cardiac Surgery; LM, left main artery; LAD, left anterior descending artery; LCX, left circumflex artery; RCA, right coronary artery*.

During the median follow-up of 31 months (IQR: 31–36 months), 353 patients developed at least one primary endpoint event, which was found in 80 (14.1%) patients from the T1 group, 121 (20.9%) from the T2 group, and 152 (26.5%) from the T3 group. The number and percentage of each component of the primary endpoint were as follows: 44 (2.5%) deaths, 49 (2.8%) MIs, 25 (1.4%) strokes, and 287 (16.3%) unplanned repeated revascularizations. Detailed clinical outcomes among patients with MHR tertiles are shown in Table 2. As shown in Supplementary Table 1, patients with the primary endpoint had higher levels of MHR, blood pressure, monocyte count, neutrophil count, NLR, hs-CRP, total cholesterol, triglycerides, LDL-C, FPG, glycosylated hemoglobin, and cTnI, but had lower LVEF and HDL-C. Those patients also had higher rate of diabetes, previous MI, prior PCI, CKD, PAD, and heart failure. In terms of the angiographic findings, patients with the primary endpoint were associated with higher rate of left-main and/or multivessel lesions, chronic total occlusion, lesions longer than 20 mm, bifurcation or trifurcation lesions and had higher SYNTAX score but lower rate of complete revascularization.

**Table 2:** Adverse cardiovascular events according to MHR tertiles during follow-up.

**Adverse cardiovascular events**	**T1: < 7.7**	**T2: 7.7–11.3**	**T3: > 11.3**	***P*** **-value**
Primary endpoint, *n* (%)	80 (14.1)	121 (20.9)	152 (26.5)	<0.001
Overall death, *n* (%)	6 (1.1)	20 (3.5)	18 (3.1)	0.021
Non-fatal MI, *n* (%)	9 (1.6)	15 (2.6)	25 (4.5)	0.017
Non-fatal stroke, *n* (%)	6 (1.1)	8 (1.4)	11 (1.9)	0.474
Unplanned repeat revascularization, *n* (%)	67 (11.8)	96 (16.6)	124 (21.6)	<0.001

*The primary endpoint was defined as the composite of overall death, non-fatal stroke, non-fatal MI, and unplanned repeat revascularization. MI, myocardial infarction*.

**Figure 1:**
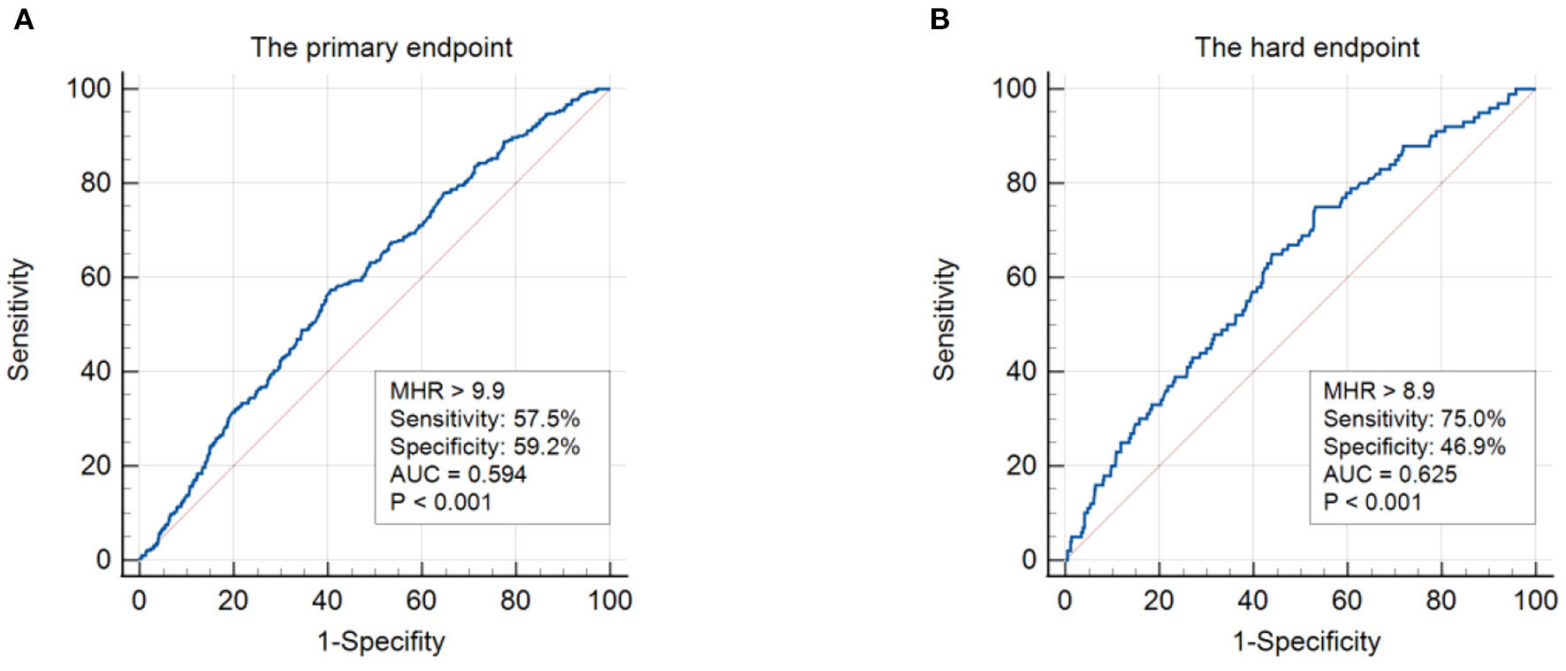
ROC analysis showing the cut-off values of MHR to predict the primary endpoint **(A)** and the hard endpoint **(B)**. The primary endpoint was defined as the composite of overall death, non-fatal stroke, non-fatal MI, and unplanned repeat revascularization. The hard endpoint was defined as the composite of cardiovascular death, non-fatal stroke, and non-fatal MI. ROC, receiver operating curve; MHR, monocyte to HDL ratio; AUC, area under the curve; MI, myocardial infarction.

**Figure 2:**
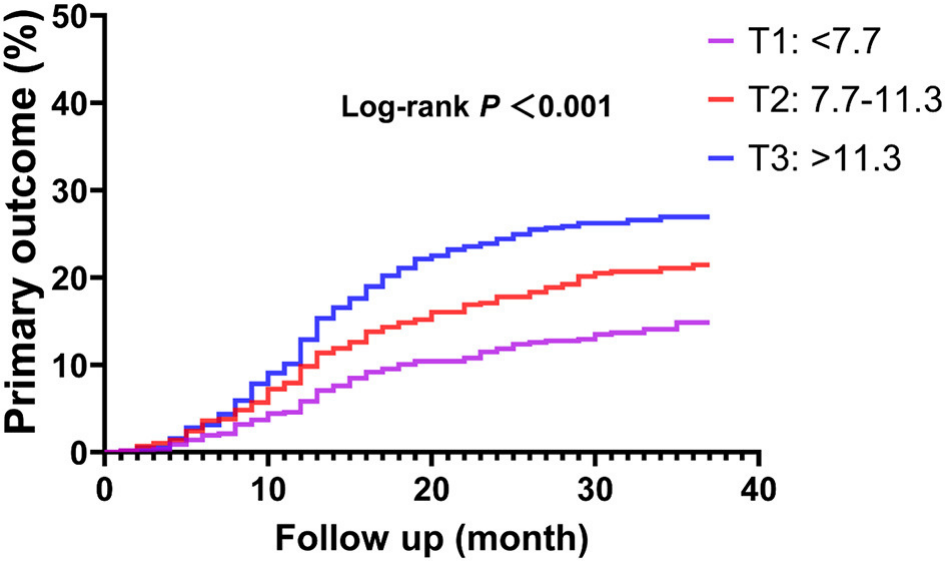
Kaplan-Meier curve of the primary endpoint stratified by the MHR tertiles. The primary endpoint was defined as the composite of overall death, non-fatal stroke, non-fatal MI, and unplanned repeat revascularization. MI, myocardial infarction.

**Figure 3:**
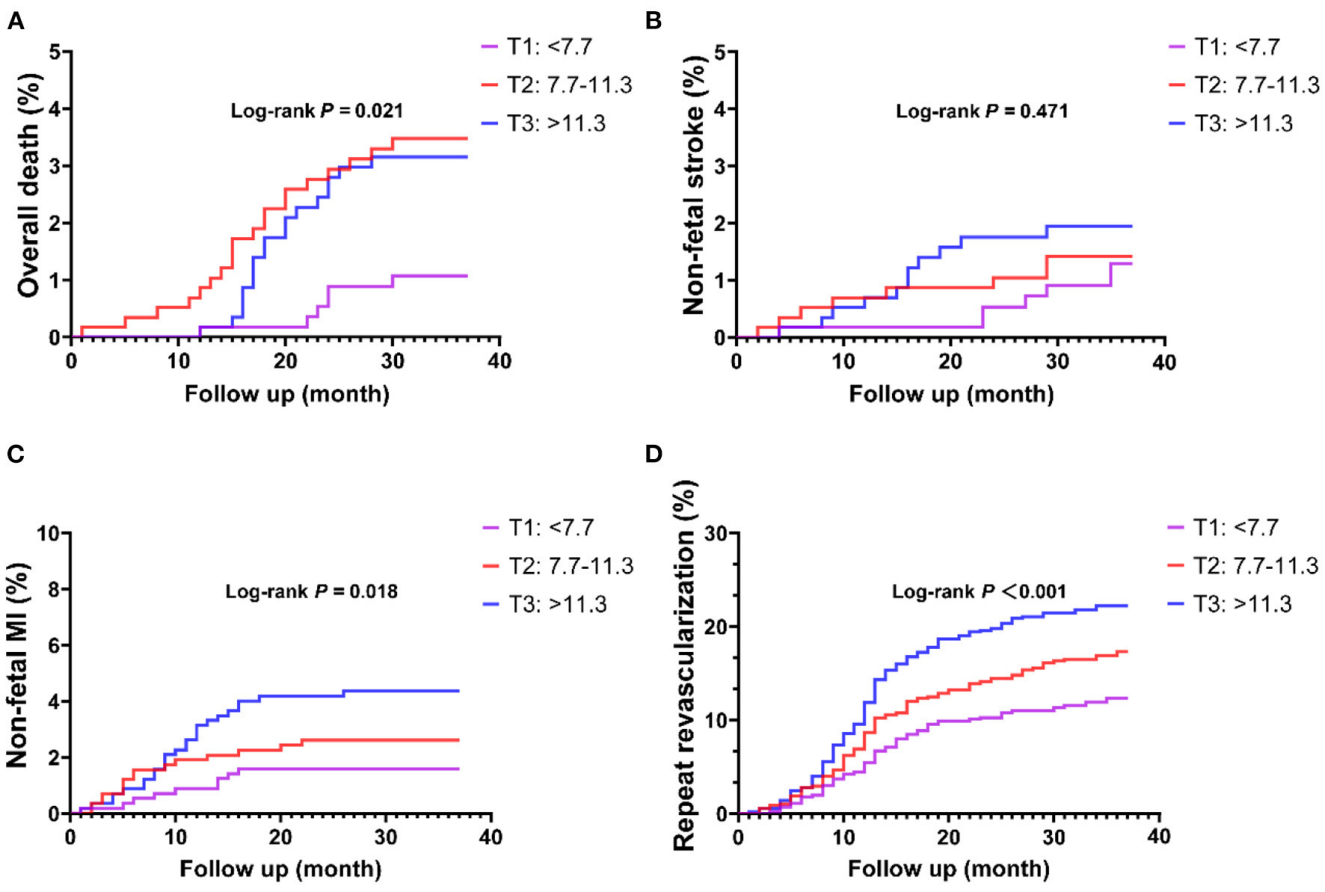
Kaplan-Meier curves of each component of the primary endpoint stratified by the MHR tertiles. **(A)** Overall death; **(B)** non-fatal stroke; **(C)** non-fatal MI; **(D)** repeat revascularization. MI, myocardial infarction.

In ROC analysis, a MHR cut-off value of 9.9 had 57.5% sensitivity and 59.2% specificity for prediction of the primary endpoint (AUC = 0.594, 95% CI: 0.562–0.627, *P* < 0.001; Figure 1A). Moreover, a MHR cut-off value of 8.9 had 75.0% sensitivity and 46.9% specificity for prediction of the hard endpoint (AUC = 0.625, 95% CI: 0.571–0.680, *P* < 0.001; Figure 1B). Kaplan-Meier survival analysis showed that the cumulative incidence of the primary endpoint increased with higher MHR tertiles (log-rank test, *P* < 0.001; Figure 2). The difference in the cumulative incidence of the primary endpoint was mainly driven by an increase in overall death (log-rank test, *P* = 0.021), non-fatal MI (log-rank test, *P* = 0.018), and unplanned repeat revascularization (log-rank test, *P* < 0.001). However, the incidence of non-fatal stroke among the MHR tertiles were similar (log-rank test, *P* = 0.471; Figure 3).

**Figure 4:**
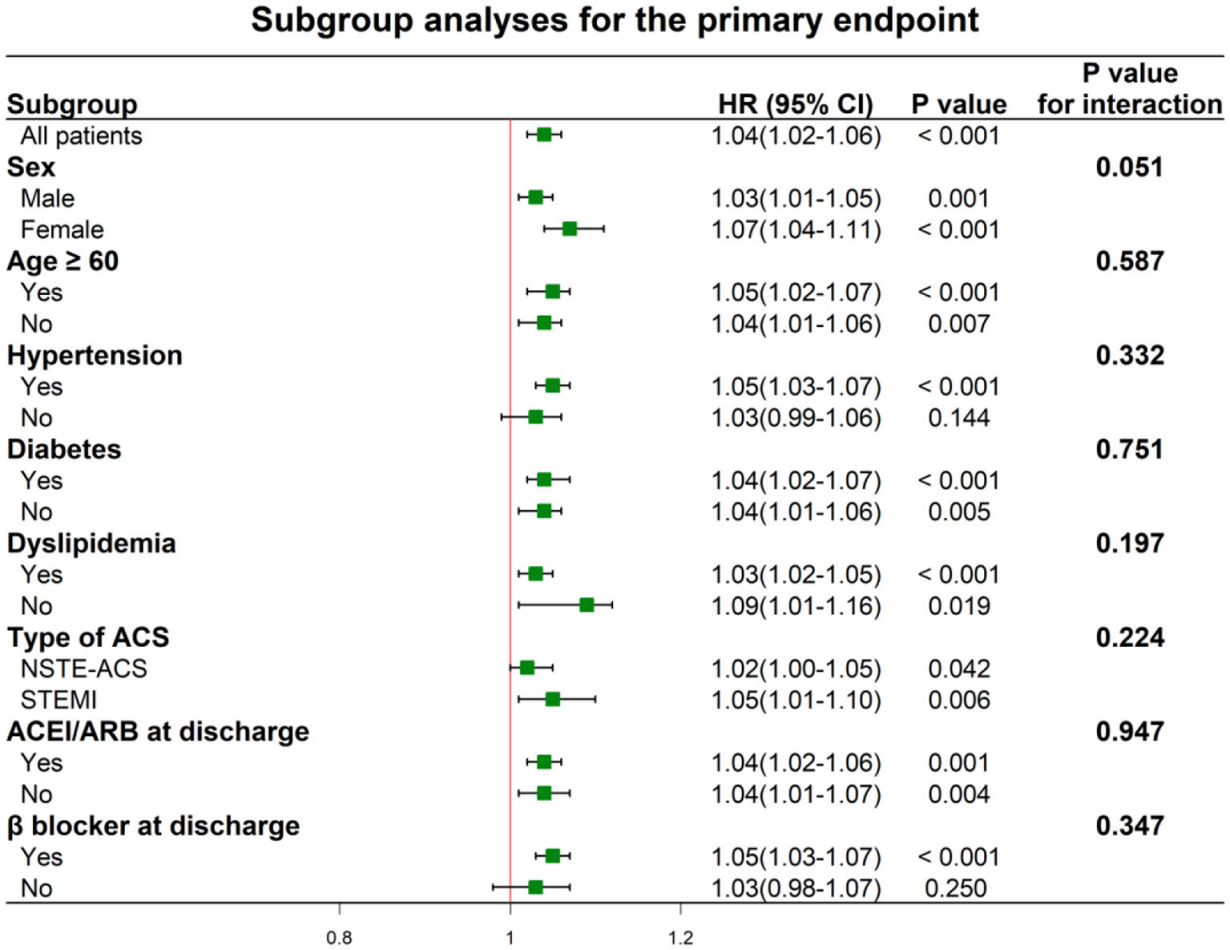
Hazard ratios for the primary endpoint according to the subgroups. ACS, acute coronary syndrome; NSTE-ACS, non ST-segment elevation acute coronary syndrome; STEMI, ST-segment elevation myocardial infarction; ACEI, angiotensin converting enzyme inhibitor; ARB, angiotensin II receptor blocker.

Univariate and multivariate Cox proportional hazards regressions for the primary endpoint are shown in Table 3 and Supplementary Table 2, where the MHR were considered as categorical variable and continuous variable, respectively. In the univariate analyses, patients in the higher MHR tertiles were at higher risk of the primary endpoint [HR: 1.54 (95% CI: 1.16–2.04) for T2 and 2.03 (95% CI: 1.55–2.67) for T3]. After adjusting for other confounding factors, MHR across the tertiles could independently predict the occurrence of primary endpoint [adjusted HR: 1.45 [95% CI: 1.08–1.95) for T2 and 1.76 (95% CI: 1.30–2.40) for T3]. The incidence of the primary endpoint was monotonically increased across the MHR tertiles (*P* for trend < 0.001). When considering as a continuous variable, MHR was associated with an HR of 1.04 (95% CI: 1.02–1.06, *P* < 0.001) in the univariate analysis and with an HR of 1.03 (95% CI: 1.01–1.05, *P* = 0.004) in the multivariate analysis. In terms of the hard endpoint, the multivariate Cox proportional hazards regression analysis adjusted for multiple confounding factors revealed a hazard ratio for the hard endpoint of 2.34 (95% CI 1.27–4.33; *P* for trend = 0.023) when the highest and lowest MHR tertiles were compared. Subgroup analyses of MHR as a continuous variable for the primary endpoint were performed according to sex, age, hypertension, diabetes, dyslipidemia, type of ACS, and medications at discharge (Figure 4). One unit increase of MHR had a significant predictive role for different subgroups regardless of male or female, age ≥ or <60 years, hypertension or not, diabetes or not, dyslipidemia or not, STEMI or NSTE-ACS (unstable angina + NSTEMI), ACEI/ARBs use or not at discharge, β-blockers use or not at discharge (all *P* for interaction > 0.05).

**Table 3:** Univariate and multivarite cox proportional hazards analyses for the primary endpoint according to the MHR tertiles.

**Variables**	**Univariate analysis**			**Multivariate analysis**		
	**HR**	**95% CI**	***P*** **-value**	**HR**	**95% CI**	***P*** **-value**
**MHR tertiles**						
T1	Reference			Reference		
T2	1.54	1.16–2.04	0.003	1.45	1.08–1.95	0.013
T3	2.03	1.55–2.67	<0.001	1.76	1.30–2.40	<0.001
hs-CRP	1.03	1.02–1.05	<0.001	1.02	1.00–1.04	0.03
Sex	1.03	0.80–1.32	0.848	0.70	0.52–0.93	0.015
Smoking	1.15	0.93–1.41	0.200	1.24	0.97–1.59	0.089
Hypertension	1.06	0.85–1.32	0.592	1.07	0.84–1.36	0.589
Diabetes	1.51	1.22–1.86	<0.001	1.31	1.05–1.63	0.016
Dyslipidemia	1.32	1.00–1.75	0.051	0.98	0.72–1.32	0.876
Previous MI	1.55	1.23–1.97	<0.001	1.12	0.85–1.47	0.418
Previous PCI	1.59	1.26–2.00	<0.001	1.44	1.09–1.91	0.01
previous CVA	1.09	0.71–1.68	0.703	0.62	0.39–0.97	0.035
PAD	2.74	2.12–3.54	<0.001	2.28	1.70–3.06	<0.001
**Type of ACS**						
Unstable angina	Reference			Reference		
NSTEMI	1.24	0.92–1.67	0.155	1.15	0.78–1.71	0.489
STEMI	1.06	0.78–1.45	0.717	1.24	0.76–2.02	0.396
GRACE risk score	1.00	1.00–1.01	0.05	1.00	0.99–1.00	0.317
SYNTAX score	1.03	1.03–1.04	<0.001	1.02	1.01–1.03	0.005
Complete revascularization	0.43	0.35–0.53	<0.001	0.54	0.42–0.68	<0.001
Aspirin at discharge	0.24	0.13–0.46	<0.001	0.54	0.42–0.68	0.031
ACEI/ARBs at discharge	1.12	0.91–1.38	0.287	0.95	0.76–1.20	0.675
β-blockers at discharge	0.76	0.61–0.95	0.016	0.61	0.49–0.77	<0.001

*HR, hazard ratio; CI., confidential interval. Other abbreviations as in Table 1*.

The addition of MHR had an incremental effect on the predictive ability of the GRACE risk score for the primary endpoint (C-statistic: GRACE risk score + MHR vs GRACE risk score: 0.590 vs. 0.525, *P* < 0.001; cNRI: 0.136, *P* < 0.001; IDI: 0.006, *P* < 0.001; Table 4). Moreover, the addition of MHR could significantly increase the C-statistics of the GRACE risk score for the composite of death or MI and the composite of death, stroke, or MI (GRACE risk score + MHR vs. GRACE score: 0.648 vs. 0.605, *P* = 0.003, and 0.667 vs. 0.622, *P* = 0.023, respectively).

**Table 4:** Model performance after the addition of MHR to the GRACE risk score for predicting clinical outcomes.

	**C-statistic**	***P*** **-value**	**cNRI (95% CI)**	***P*** **-value**	**IDI(95% CI)**	***P*** **-value**
**Primary endpoint**						
GRACE score	0.525	<0.001	Ref	<0.001	Ref	<0.001
GRACE score + MHR	0.590		0.136 (0.062–0.195)		0.006 (0.001–0.018)	
**Death or MI**						
GRACE score	0.605	0.003	Ref	0.218	Ref	0.832
GRACE score + MHR	0.648		0.082 (−0.035–0.200)		0.000 (−0.001–0.008)	
**Death, stroke, or MI**						
GRACE score	0.622	0.023	Ref	0.297	Ref	0.495
GRACE score + MHR	0.667		0.075 (−0.032–0.175)		0.001 (−0.001–0.013)	

*MHR, monocyte to HDL-C ratio; GRACE, Global registry of acute coronary events; MI, myocardial infarction; cNRI, category-free continuous net reclassification improvement; IDI, integrated discrimination improvement; CI, confidential interval*.

## Discussion

The present study investigated the relationship between MHR and adverse CV outcomes in patients with ACS undergoing PCI. The results showed that even after adjusting for as many confounding factors as possible, MHR remained significantly associated with the primary endpoint. Furthermore, the addition of MHR significantly improved the predictive ability of the GRACE risk score for adverse CV outcomes.

Atherosclerosis, as the main cause of CAD, is considered as an inflammatory disease. Inflammatory responses have been shown to be significantly enhanced in both obstructive and non-obstructive acute myocardial infarction patients accompanied with hyperglycemia (22). The inflammatory process is dominated by monocyte-derived macrophages in the early stage of plaque formation. Tissue-infiltrating macrophages eventually become the primary immune cells of plaque, where they take up cholesterol and store lipids in the form of small droplets, resulting in a unique morphology called foam cells (23). Monocyte recruitment governs the expansion of plaque formation, and the inhibition of monocyte infiltration and differentiation can attenuate early atherogenesis (24). Monocyte transmigration is induced by LDL but inhibited by HDL (25). Statins that used to lower LDL-C can reduce the number of monocyte-derived macrophages and the levels of C-reactive protein, eventually leading to plaque regression (26). Additionally, monocytes are associated with plaque rupture by secreting a variety of enzymes such as matrix metalloproteinases which are involved in the breakdown of the arterial extracellular matrix (27). HDL has been demonstrated to protect against plaque formation via promoting the efflux of cholesterol from cells and decreasing cholesterol levels (28). Moreover, HDL has anti-inflammation and anti-oxidation properties by reducing lipid peroxidation and deposition, and minimizing the accumulation of foam cells in the artery wall (29). The intrinsic relationship between monocytes and HDL in the development of atherosclerosis suggests that the combination of monocytes and HDL-C (presented as MHR) may better reflect the inflammatory process of atherosclerosis than either alone.

Indeed, MHR reflects both immune and metabolic status and is associated with pathological processes in many diseases (30–34). In patients with atherosclerotic CV disease mainly characterized by inflammation and lipid deposition, MHR has a significant prognostic role. It was reported that a higher MHR was associated with a worse prognosis in CAD patients who underwent PCI (14). MHR was shown to be an independent predictor of in-stent restenosis and adverse CV outcomes in patients with STEMI (35–37). Also, a higher MHR was demonstrated to be independently associated with a higher risk of intracoronary thrombus burden and no-reflow phenomenon (38, 39). Similar to these studies, we also found MHR was an independent predictor of adverse CV outcomes in patients with ACS undergoing PCI. Of note, there was a borderline differential effect of MHR across sex in our study. MHR seemed to have better predictive value of cardiovascular outcomes in female patients than in male patients. As we known, clinical outcomes from ACS are worse for women than for men (40). In our study, the correlation of MHR with hs-CRP was more significant in female patients (*r* = 0.424, *P* < 0.001) than in male patients (*r* = 0.418, *P* < 0.001). The hs-CRP is considered an ideal indicator of systemic inflammation and has been shown to be strongly associated with poor prognosis (41). This may be one reason why there was a borderline differential effect of MHR across sex. However, prospective studies with large sample sizes and sufficient statistical power are needed to confirm sex differences in MHR prediction of adverse cardiovascular events.

The GRACE risk score derived from an international registry involving more than 100,000 patients in 30 countries has been widely used to predict in-hospital and long-term outcomes in ACS patients. Many studies have confirmed the short- and long-term predictive value of the GRACE risk score (42, 43). However, inflammatory and lipid biomarkers were not taken into consideration in the GRACE risk score. In terms of inflammatory biomarkers, previous studies have shown that C-reactive protein has a weak correlation with the GRACE risk score and the addition of C-reactive protein can improve the predictive ability of the GRACE risk score (44). Also, the introduction of neutrophil counts or neutrophil to lymphocyte ratio can increase the predictive value of the GRACE risk score (45). Remarkably, monocyte counts were shown to be correlated with the GRACE risk score (46). Considering lipid biomarkers, the addition of LDL-C or lipoprotein(a) has been demonstrated to improve the predictive ability of the GRACE risk score (47, 48). Therefore, inflammatory and lipid biomarkers can add the predictive value on top of the GRACE risk score. In the present study, we found that adjustment of the GRACE risk score by MHR (as a marker for inflammation and lipid metabolism) improved prediction of adverse CV outcomes in patients with ACS undergoing PCI.

## Limitation

Several limitations must be taken into account when interpreting the results of our study. First, the present study was only a single-center observational study, so the effects of unmeasured and undetected confounding variables cannot be excluded. Second, the data of this study were limited to the Chinese population, so the ethnic difference cannot be eradicated. Third, both monocytes and HDL-C were measured at admission; however, values measured at discharge or changes during follow-up may be even more predictive.

## Conclusion

MHR was independently and significantly associated with adverse CV outcomes and improved the predictive ability of the GRACE risk score in ACS patients who underwent PCI. Clinical trials are needed to determine whether medical management optimization based on the MHR reduces the risk of subsequent CV events.

## Data Availability Statement

The raw data supporting the conclusions of this article will be made available by the authors, without undue reservation.

## Ethics Statement

The studies involving human participants were reviewed and approved by local Ethics Committee of Beijing Anzhen Hospital, Capital Medical University. Written informed consent for participation was not required for this study in accordance with the national legislation and the institutional requirements.

## Author Contributions

XM and KH analyzed the data and drafted the manuscript. XM, DS, and YZ designed the study and revised the manuscript. All authors contributed to the acquisition of data and final approval of the version to be published.

## Funding

This work was supported by National Key Research and Development Program of China (2017YFC0908800), China Postdoctoral Science Foundation (2021M692253), Beijing Postdoctoral Research Foundation (2021-ZZ-023), and Beijing Municipal Administration of Hospitals Mission Plan (SML20180601).

## Conflict of Interest

The authors declare that the research was conducted in the absence of any commercial or financial relationships that could be construed as a potential conflict of interest.

## Publisher's Note

All claims expressed in this article are solely those of the authors and do not necessarily represent those of their affiliated organizations, or those of the publisher, the editors and the reviewers. Any product that may be evaluated in this article, or claim that may be made by its manufacturer, is not guaranteed or endorsed by the publisher.

## Abbreviations

ACEI: angiotensin converting enzyme inhibitor
ACS: acute coronary syndrome
ARB: angiotensin II receptor blocker
BMI: body mass index
CAD: coronary artery disease
CI: confidence interval
CKD: chronic kidney disease
cNRI: continuous net reclassification improvement
CV: cardiovascular
FPG, fasting plasma glucose, GRACE: Global Registry of Acute Coronary Events
HDL: high-density lipoprotein
HDL-C: high-density lipoprotein-cholesterol
HF: heart failure
HR: hazard ratio
hs-CRP: high-sensitivity C-reactive protein
IDI: integrated discrimination improvement
IQR: interquartile range
LDL-C: low-density lipoprotein-cholesterol
LVEF: left ventricular ejection fraction
MHR: monocyte to HDL-C ratio
MI: myocardial infarction
NLR: neutrophil to lymphocyte ratio
NSTEMI: non-ST segment elevation myocardial infarction
NSTE-ACS: non-ST segment elevation acute coronary syndrome
PAD: peripheral artery disease
PCI: percutaneous coronary intervention
ROC: Receiver operating characteristic
SD: standard deviation
STEMI: ST segment elevation myocardial infarction
SYNTAX: SYNergy between percutaneous coronary intervention with TAXus and cardiac surgery
UA: unstable angina.
