# Supplementary material for: Adjustment of the GRACE Risk Score by Monocyte to High-Density Lipoprotein Ratio Improves Prediction of Adverse Cardiovascular Outcomes in Patients With Acute Coronary Syndrome Undergoing Percutaneous Coronary Intervention
Source: Front Cardiovasc Med. 2022 Jan 26;8:755806. doi: 10.3389/fcvm.2021.755806 (PMC8826569; doi:10.3389/fcvm.2021.755806)
Supplement: Supplementary file 1 [file Table_1.DOCX]

Supplemental Table 1. Baseline characteristics of the study population according to the primary endpoint

| Variables | Without event (1367) | With event (353) | P value |
| --- | --- | --- | --- |
| MHR | 10.0±5.0 | 11.3±4.9 | <0.001 |
| MHR tertiles |  |  |  |
| T1, n (%) | 486 (35.6) | 80 (22.7) | <0.001 |
| T2, n (%) | 459 (33.6) | 121 (34.3) |  |
| T3, n (%) | 422 (30.9) | 152 (43.1) |  |
| Age (years) | 59.7±10.3 | 60.4±10.7 | 0.253 |
| Male sex, n (%) | 1046 (76.5) | 273 (77.3) | 0.746 |
| BMI (kg/m^2^) | 25.7±3.1 | 25.4±3.2 | 0.103 |
| SBP (mmHg) | 129.4±16.4 | 131.9±17.1 | 0.010 |
| DBP (mmHg) | 76.7±10.6 | 73.4±10.6 | <0.001 |
| Risk factors |  |  |  |
| Smoking, n (%) | 594 (43.5) | 166 (47.0) | 0.228 |
| Hypertension, n (%) | 866 (63.4) | 229 (64.9) | 0.596 |
| Diabetes, n (%) | 597 (43.7) | 193 (54.7) | <0.001 |
| Dyslipidemia, n (%) | 1080 (79.0) | 295 (83.6) | 0.056 |
| Previous MI, n (%) | 237 (17.3) | 93 (26.4) | <0.001 |
| Previous PCI, n (%) | 244 (17.9) | 97 (27.5) | <0.001 |
| Previous CVA, n (%) | 78 (5.7) | 22 (6.2) | 0.706 |
| CKD, n (%) | 28 (2.0) | 21 (5.9) | <0.001 |
| PAD, n (%) | 102 (7.5) | 74 (21.0) | <0.001 |
| Heart failure, n (%) | 80 (5.9) | 40 (11.3) | <0.001 |
| LVEF (%) | 65 (60-68) | 62 (58-67) | <0.001 |
| Clinical presentation |  |  |  |
| Unstable angina, n (%) | 1022 (74.8) | 254 (72.0) | 0.283 |
| NSTEMI, n (%) | 168 (12.3) | 52 (14.7) | 0.221 |
| STEMI, n (%) | 177 (13.0) | 47 (13.3) | 0.855 |
| GRACE risk score | 103.0±38.3 | 107.5±41.1 | 0.053 |
| Laboratory results |  |  |  |
| Monocyte (×10^6^/μl) | 350 (280-450) | 390 (310-485) | <0.001 |
| Neutrophil count (×10^6^/μl) | 3.89 (3.15-4.76) | 4.41 (3.55-5.39) | <0.001 |
| Lymphocyte count (×10^6^/μl) | 1.76 (1.43-2.19) | 1.67 (1.37-2.12) | 0.081 |
| NLR | 2.19 (1.67-2.88) | 2.56 (1.89-3.44) | <0.001 |
| hs-CRP (mg/L) | 1.23 (0.57-3.13) | 2.13 (0.93-5.27)  ( | <0.001 |
| Total cholesterol (mg/dl) | 158.9±38.2 | 165.6±38.3 | 0.003 |
| LDL-C (mg/dl) | 93.3±31.5 | 98.5±30.3 | 0.006 |
| HDL-C (mg/dl) | 40.4±9.3 | 38.2±8.1 | <0.001 |
| Triglycerides (mg/dl) | 124.9 (87.7-178.0) | 142.6 (98.3-202.4) | <0.001 |
| FPG (mg/dl) | 111.4±27.8 | 125.8±39.3 | <0.001 |
| Glycosylated hemoglobin (%) | 6.0 (5.5-7.0) | 6.4 (5.7-7.4) | <0.001 |
| cTnI (ng/ml) | 0.00 (0.00-0.01) | 0.01 (0.00-0.04) | <0.001 |
| Admission medical therapy |  |  |  |
| Aspirin, n (%) | 997 (72.9) | 265 (75.1) | 0.418 |
| P2Y12 inhibitors, n (%) | 552 (40.4) | 149 (42.2) | 0.533 |
| Statins, n (%) | 977 (71.5) | 261 (73.9) | 0.357 |
| ACEI/ARBs, n (%) | 379 (27.7) | 112 (31.7) | 0.138 |
| β-blockers, n (%) | 520 (38.0) | 119 (33.7) | 0.134 |
| Angiographic findings |  |  |  |
| Left-main and/or multivessel disease, n (%) | 1138 (83.2) | 320 (90.7) | <0.001 |
| Chronic total occlusion, n (%) | 274 (20.0) | 89 (25.2) | 0.034 |
| Lesions with length >20mm, n (%) | 683 (50.0) | 219 (62.0) | <0.001 |
| Bifurcation or trifurcation lesions, n (%) | 1014 (74.2) | 284 (80.5) | 0.015 |
| SYNTAX score | 19 (12-27) | 25 (17-33) | <0.001 |
| Procedural results |  |  |  |
| Target vessel-LM, n (%) | 43 (3.1) | 14 (4.0) | 0.443 |
| Target vessel-LAD, n (%) | 302 (22.1) | 93 (26.3) | 0.090 |
| Target vessel-LCX, n (%) | 168 (12.3) | 55 (15.6) | 0.101 |
| Target vessel-RCA, n (%) | 237 (17.3) | 86 (24.4) | 0.003 |
| Complete revascularization, n (%) | 905 (66.2) | 152 (43.1) | <0.001 |
| Prescription at discharge |  |  |  |
| Aspirin, n (%) | 1361 (99.6) | 343 (97.2) | <0.001 |
| Clopidogrel, n (%) | 1260 (92.2) | 319 (90.4) | 0.271 |
| Ticagrelor, n (%) | 107 (7.8) | 34 (9.6) | 0.271 |
| Statins, n (%) | 1367 (100) | 353 (100) | NA |
| ACEI/ARBs, n (%) | 649 (47.5) | 179 (50.7) | 0.279 |
| β-blockers, n (%) | 981 (71.8) | 230 (65.2) | 0.015 |

Abbreviations as in Table 1.
