# Supplementary material for: Adjustment of the GRACE Risk Score by Monocyte to High-Density Lipoprotein Ratio Improves Prediction of Adverse Cardiovascular Outcomes in Patients With Acute Coronary Syndrome Undergoing Percutaneous Coronary Intervention
Source: Front Cardiovasc Med. 2022 Jan 26;8:755806. doi: 10.3389/fcvm.2021.755806 (PMC8826569; doi:10.3389/fcvm.2021.755806)
Supplement: Supplementary file 2 [file Table_2.DOCX]

Supplemental Table 2. Univariate and multivariate Cox proportional hazards models for the primary endpoint when considering MHR as a continuous variable

| Variables | Univariate analysis | |  | Multivariate analysis | |  |
| --- | --- | --- | --- | --- | --- | --- |
|  | HR | 95%CI | P value | HR | 95%CI | P value |
| MHR | 1.04 | 1.02-1.06 | <0.001 | 1.027 | 1.01-1.05 | 0.015 |
| hs-CRP | 1.03 | 1.02-1.05 | <0.001 | 1.02 | 1.00-1.04 | 0.068 |
| Sex | 1.03 | 0.80-1.31 | 0.848 | 0.72 | 0.54-0.97 | 0.029 |
| Smoking | 1.15 | 0.93-1.41 | 0.200 | 1.29 | 1.01-1.65 | 0.045 |
| Hypertension | 1.06 | 0.85-1.32 | 0.592 | 1.07 | 0.84-1.35 | 0.608 |
| Diabetes | 1.51 | 1.22-1.86 | <0.001 | 1.33 | 1.07-1.65 | 0.012 |
| Dyslipidemia | 1.32 | 1.00-1.75 | 0.051 | 1.01 | 0.75-1.36 | 0.944 |
| Previous MI | 1.55 | 1.23-1.97 | <0.001 | 1.16 | 0.88-1.52 | 0.289 |
| Previous PCI | 1.59 | 1.26-2.00 | <0.001 | 1.44 | 1.09-1.91 | 0.010 |
| Previous CVA | 1.09 | 0.71-1.68 | 0.703 | 0.65 | 0.41-1.02 | 0.058 |
| PAD | 2.74 | 2.12-3.54 | <0.001 | 2.29 | 1.70-3.08 | <0.001 |
| Type of ACS |  |  |  |  |  |  |
| Unstable angina | Reference | | | Reference | | |
| NSTEMI | 1.24 | 0.92-1.67 | 0.155 | 1.19 | 0.81-1.76 | 0.392 |
| STEMI | 1.06 | 0.78-1.45 | 0.717 | 1.31 | 0.81-2.14 | 0.272 |
| GRACE risk score | 1.00 | 1.00-1.01 | 0.05 | 1.00 | 0.99-1.00 | 0.223 |
| SYNTAX score | 1.03 | 1.03-1.04 | <0.001 | 1.02 | 1.01-1.03 | 0.005 |
| Complete revascularization | 0.43 | 0.35-0.53 | <0.001 | 0.54 | 0.42-0.68 | <0.001 |
| Aspirin at discharge | 0.24 | 0.13-0.46 | <0.001 | 0.44 | 0.23-0.85 | 0.014 |
| ACEI/ARBs at discharge | 1.12 | 0.91-1.38 | 0.287 | 0.96 | 0.76-1.21 | 0.718 |
| β-blockers at discharge | 0.76 | 0.61-0.95 | 0.016 | 0.62 | 0.50-0.78 | <0.001 |

Abbreviations as in Table 3.
